# Potential to enhance telephone cardiopulmonary resuscitation with improved instructions - findings from a simulation-based manikin study with lay rescuers

**DOI:** 10.1186/s12873-023-00810-0

**Published:** 2023-04-01

**Authors:** Carlos Ramon Hölzing, Peter Brinkrolf, Camilla Metelmann, Bibiana Metelmann, Klaus Hahnenkamp, Mina Baumgarten

**Affiliations:** Department of Anaesthesiology, University Medicine Greifswald, Ferdinand-Sauerbruch Straße 1, 17475 Greifswald, Germany

**Keywords:** Cardiopulmonary resuscitation, CPR, Heart arrest, Out-of-Hospital cardiac arrest, Emergency medical dispatch, Bystander resuscitation, Life support care, Dispatcher-assisted CPR, Telephone CPR

## Abstract

**Background:**

Telephone-Cardiopulmonary Resuscitation (T-CPR) significantly increases rate of bystander resuscitation and improves patient outcomes after out-of-hospital cardiac arrest (OHCA). Nevertheless, securing correct execution of instructions remains a difficulty. ERC Guidelines 2021 recommend standardised instructions with continuous evaluation. Yet, there are no explicit recommendations on a standardised wording of T-CPR in the German language. We investigated, whether a modified wording regarding check for breathing in a German T-CPR protocol improved performance of T-CPR.

**Methods:**

A simulation study with 48 OHCA scenarios was conducted. In a non-randomised trial study lay rescuers were instructed using the real-life-CPR protocol of the regional dispatch centre and as the intervention a modified T-CPR protocol, including specific check for breathing (head tilt-chin lift instructions). Resuscitation parameters were assessed with a manikin and video recordings.

**Results:**

Check for breathing was performed by 64.3% (*n* = 14) of the lay rescuers with original wording and by 92.6% (*n* = 27) in the group with modified wording (*p* = 0.035). In the original wording group the head tilt-chin manoeuvre was executed by 0.0% of the lay rescuers compared to 70.3% in the group with modified wording (*p* < 0.001). The average duration of check for breathing was 1 ± 1 s in the original wording group and 4 ± 2 s in the group with modified wording (*p* < 0.001). Other instructions (e.g. check for consciousness and removal of clothing) were well performed and did not differ significantly between groups. Quality of chest compression did not differ significantly between groups, with the exception of mean chest compression depth, which was slightly deeper in the modified wording group.

**Conclusion:**

Correct check for breathing seems to be a problem for lay rescuers, which can be decreased by describing the assessment in more detail. Hence, T-CPR protocols should provide standardised explicit instructions on how to perform airway assessment. Each protocol should be evaluated for practicability.

## Background

Out-of-hospital cardiac arrest (OHCA) is a worldwide common condition with a global incidence of 83.7/100,000 per year [1, 2]. Across Europe survival after OHCA varied between 5 and 30% in 2019 [3]. Immediate resuscitation by bystanders can double to triple survival rates [4–7]. To assist untrained lay rescuers in starting cardiopulmonary resuscitation (CPR), telephone-CPR (T-CPR) has been widely implemented in emergency medical services. In T-CPR, the dispatcher instructs the lay rescuer by telephone on how to perform CPR. This significantly increases the rate of bystander CPR, which improves patient outcomes [8, 9]. European Resuscitation Council (ERC) Guidelines 2021 recommend the use of standardised algorithms in telephone resuscitation, which should be continuously evaluated [10]. Like many other countries, Germany does not have a nationwide T-CPR protocol [11]. A wide variety of protocols exists and quality of implementation is evaluated only sporadically [12].

Instructions to check for breathing prove to be particularly difficult in T-CPR protocols [13, 14]. The aim of this study was to analyse, whether a specified wording in the T-CPR protocol (instructing head tilt-chin lift) improves airway assessment and quality of resuscitation parameters in a non-randomised trial study. The primary hypothesis of the study was that specifying the standard for check for breathing with head tilt-chin lift instructions leads to a significant improvement in performance of check for breathing. The secondary hypothesis was that the time interval from beginning the emergency call to applying first chest compression would be longer with the modified check for breathing instructions.

## Methods

### Study design

This controlled non-randomised trial was part of the exploratory observational project MV|LIFE|DRONE-Pilot, funded by the German Federal Ministry of Health and approved by the Ethics Committee of Greifswald University (BB124/19). In each simulation, lay rescuers were instructed with T-CPR to resuscitate.

### Participants

The lay rescuers were recruited through media as well as word-of-mouth communication. Participation in the study and the collection of anonymous data was based on obtaining written informed consent from all participants. Lay rescuers were informed they would encounter a simulated OHCA and would have to resuscitate for a maximum of 15 min. To avoid bias, no further information was given. Exclusion criteria for participation were physical limitations, resuscitation training within the past five years and no written consent. Each lay rescuer participated only once.

### Measurement and data sources

**Fig. 1:**
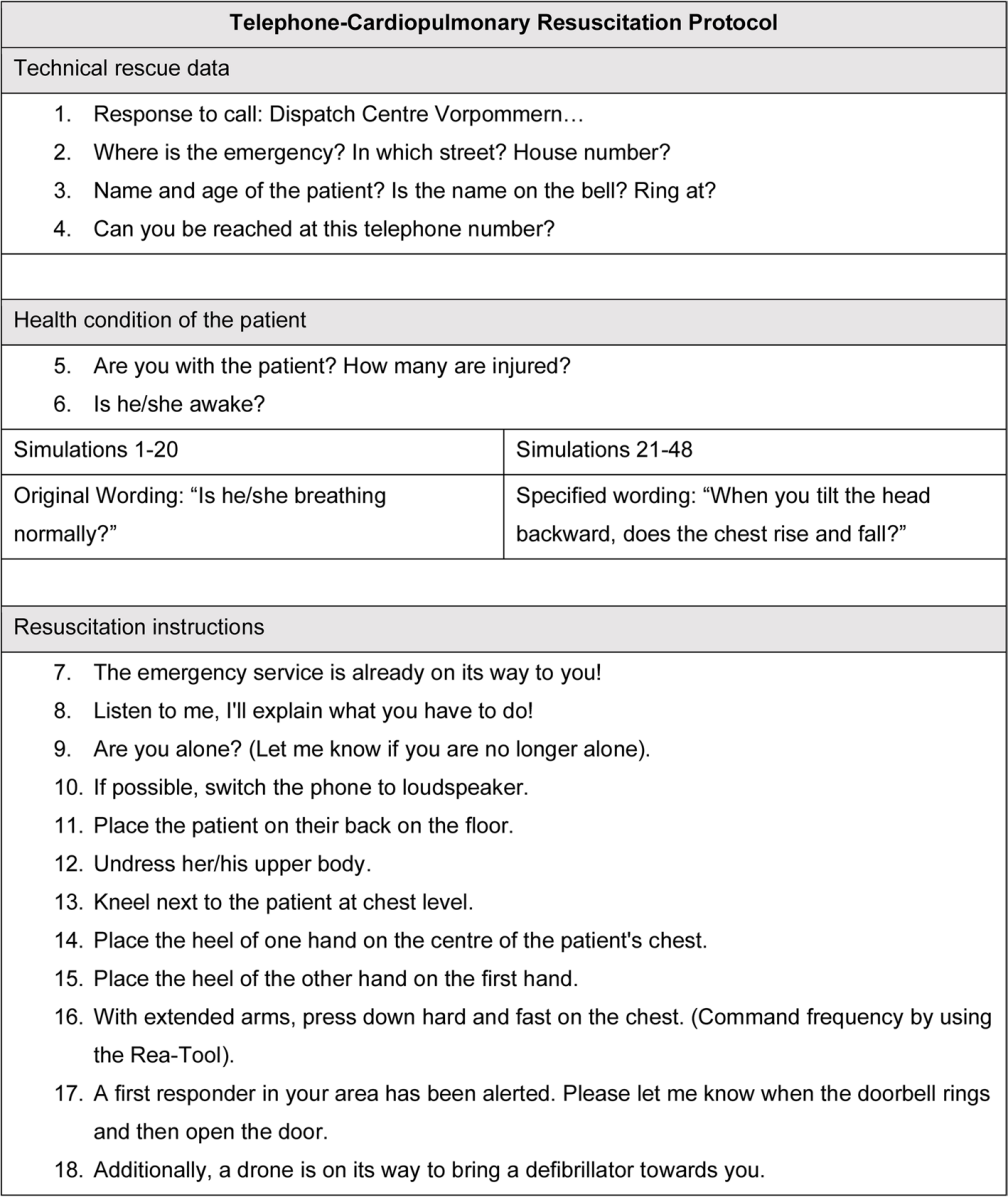
Telephone-cardiopulmonary resuscitation protocol

The simulation room was equipped with two cameras, which recorded every event in image and sound. The execution of each step of the dispatcher's instructions was measured categorically by analysing the simulation videos. To investigate the impact of the wording of check for breathing instruction the original T-CPR protocol as used by the regional dispatch centre was compared to a modified instruction on assessing breathing. The original check for breathing instruction was used in simulation 1–20 and the modified check for breathing instruction was used in simulation 21–48. In simulations 1–20 (original wording group) the wording from the protocol was: “Is he/she breathing normally? “. In simulation 21–48 (intervention group), check for breathing was specified using the following question: “When you tilt the head backward, does the chest rise and fall?”. No further parts of the protocol were changed. The entire protocol can be viewed in Fig. 1. This protocol is instructing a compression-only CPR, as recommended by ERC guidelines 2021 [10]. The T-CPR protocol of the Vorpommern-Greifswald dispatch centre was developed by iSE GmbH, Aachen, and Notruf-Training112, Mainz and is also used in 38 other dispatch centres in Germany [15].

Laerdal's Resusci Anne QCPR MK II was used as the resuscitation simulator with a wireless connected SimPad® Plus from Laerdal™. The SimPad® Plus assesses compression depth (in mm), chest compression frequency (in bpm) and chest release (0% (no release)—100% (complete release)). The resuscitation data were averaged and processed using MATLAB® R2020b (MathWorks, Natick, USA). Hand position was also evaluated using the videos and was scored as sufficient if the heel of the hand was in a 9 × 9 cm square on the centre of the chest.

### Bias

During the scenarios T-CPR was instructed by an emergency physician, who had been trained in T-CPR by a dispatcher before the first session of the study. To avoid bias the simulated dispatcher was not aware of the study hypothesis and was located in a different room. He did not see the rescuers and communication was possible only via telephone. Also, to avoid bias, the T-CPR protocol was instructed live, following real-life standards (Fig. 1).

### Study size

Sample size of our study (*n* = 48) was based on previous studies on quality of T-CPR (*n* = 30, 50, 51) [16–18]. During November and December 2019, at least 3 simulations per day were conducted on 11 simulation days.

### Statistical methods

Statistical evaluation were performed with SPSS® Statistics Version 27 (IBM Corporation Armonk, New-York, USA). Graphs were created using Prism 9® (GraphPad Software Inc., Boston, USA). Descriptive representation of nominal variables is presented with numbers (n) and valid percentages (%). Normal distribution was determined using Shapiro–Wilk test. Cluster comparison was presented using the chi-square test. If the expected frequency of data was below 5, Fisher's exact test was applied. Quantitative data were reported with mean value, standard deviation and were compared using the Mann–Whitney U test. The *p*-value for all statistical tests was set at p ≤ 0.05. Incomplete or completely missing data was excluded from the evaluation.

## Results

A total of 48 resuscitation simulations with 48 lay rescuers were conducted. Because of missing SimPad® data and discontinuation of the simulation by participants, seven simulations had to be excluded. As a result, 41 simulations were available for analysis.

The anonymous survey which followed the simulation was not completed by 3 lay rescuers. All other questionnaires (45 data sets) were included in the analysis (Table 1). As the questionnaire was anonymous no matching of the seven excluded scenarios to the corresponding questionnaires was possible.

**Table 1:** Gender and age of study participants

		Number (*n* = 45)	Percentage (%)
Gender^a^	Female	15	33.3%
	Male	30	66.7%
Age^a^	< 20	1	2.2%
	21–30	19	42.2%
	31–40	9	20.0%
	41–50	4	8.9%
	51–60	4	8.9%
	61–70	4	8.9%
	71–80	4	8.9%
	> 80	0	0.0%

Legend: ^a^ = difference caused by loss of data

### Quality of execution of T-CPR instructions

Table 2 shows the execution of T-CPR instructions in the two groups. In the intervention group with specified instructions, check for breathing was performed significantly more often (92.6% vs 64.3%, *p* = 0.035) and for a longer duration of time (4 ± 2 s vs 1 ± 1 s, *p* < 0.001). In the intervention group 19 out of 27 (70.3%) participants performed a head tilt manoeuvre, while it was performed by no participant in the original wording group (*p* < 0.001). Check for consciousness was performed by 27 (100%) lay rescuers in the intervention group and by 12 (85.7%) lay rescuers in the original wording group (*p* = 0.111). Loudspeaker function activation was performed by 25 (92.6%) lay rescuers in the intervention group and in 14 cases (100%) in the original wording group (*p* = 0.539). Removal of clothing was done by 27 (100%) lay rescuers in the intervention group and by 13 (92.9%) lay rescuers in the original wording group (*p* = 0.341). The average time from emergency call to first chest compression was 80 ± 25 s (*n* = 27) with the specified head tilt instructions and 95 ± 33 s (*n* = 14) with the original wording of the T-CPR protocol (*p* < 0.001). There was no significant difference in the position of the lay rescuers to the manikin (*p* = 1.0). In the intervention group 21 (77.8% vs. 11 (78,6%) *p* = 1.0) lay rescuers positioned themselves in a right angle to the manikin and 26 (96.3% vs. 12 (85.7%) *p* = 0.26) lay rescuers with both knees on the floor. In the intervention group 23 (85.2% vs. 11 (78.6%) *p* = 0.673) lay rescuers kept their arms straight (Table 2).

**Table 2:** Execution of the T-CPR instructions

		Check for breathing instruction		
		Original wording	Head tilt-chin lift instruction	
		Percentage %, (Number) *n* = 14	Percentage %, (Number) *n* = 27	*p*-value
Check for consciousness	No	14.3% (2)	0.0% (0)	0.111
	Yes	85.7% (12)	100.0% (27)	
Check for breathing	No	35.7% (5)	7.4% (2)	0.035
	Yes	64.3% (9)	92.6% (25)	
Head tilt-chin lift	No	100.0% (14)	29.6% (8)	< 0.001
	Yes	0.0% (0)	70.3% (19)	
Check for breathing duration (MV ± SD in sec)		1 ± 1 s	4 ± 2 s	< 0.001
Loudspeaker function activation	No	0.0% (0)	7.4% (2)	0.539
	Yes	100.0% (14)	92.6% (25)	
Removal of clothing	No	7.1% (1)	0.0% (0)	0.341
	Yes	92.9% (13)	100.0% (27)	
Rescuer position for chest compressions	Pelvis	0.0% (0)	0.0% (0)	1.000
	Head	0.0% (0)	0.0% (0)	
	Shoulder	0.0% (0)	0.0% (0)	
	Abdomen	7.1% (1)	7.4% (2)	
	Chest	92.9% (13)	92.6% (25)	
Right angle to the manikin	No	21.4% (3)	22.2% (6)	1.000
	Yes	78.6% (11)	77.8% (21)	
Both knees with ground contact	No	14.3% (2)	3.7% (1)	0.265
	Yes	85.7% (12)	96.3% (26)	
Arms stretched	No	21.4% (3)	14.8% (4)	0.673
	Yes	78.6% (11)	85.2% (23)	

### Quality of chest compressions

**Fig. 2:**
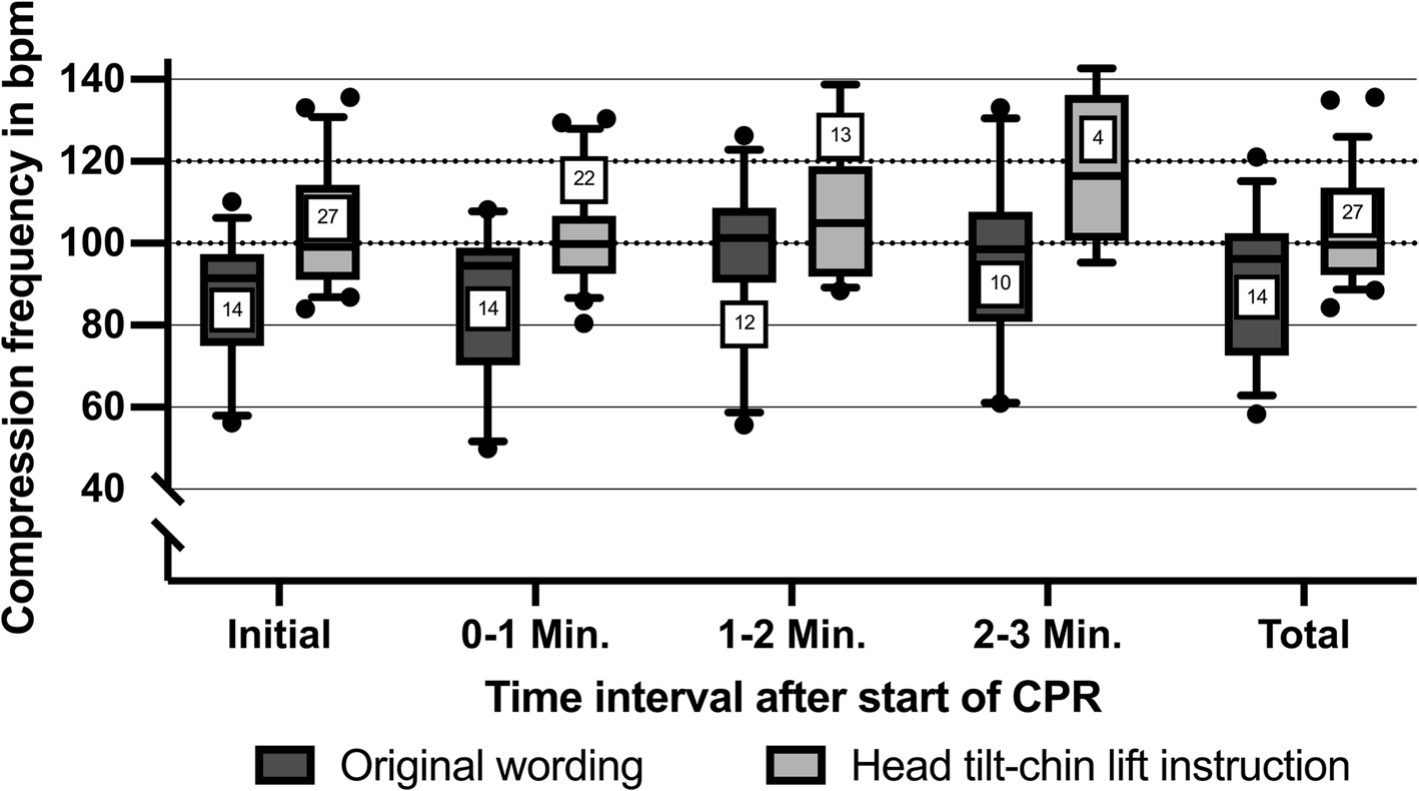
Chest compression frequency for both groups in beats per minute (numbers of participants in squares)

**Fig. 3:**
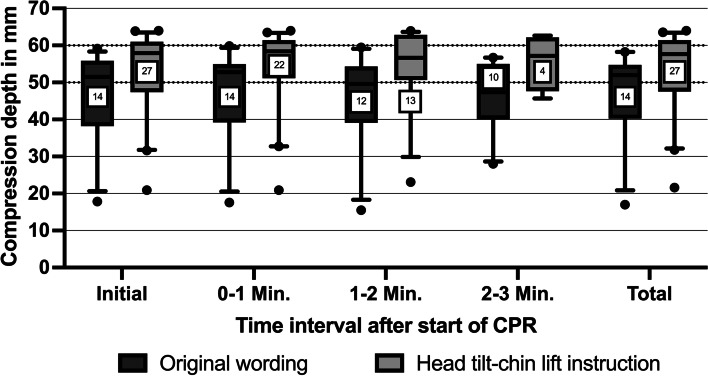
Compression depth for both groups in millimetres (numbers of participants in squares)

The mean chest compression rate did not differ significantly between groups and was 103.1 ± 14.0 bpm in the intervention group and 91.5 ± 17.2 bpm in the original wording group (*p* = 0.135) (Table 3) (Fig. 2). The average chest compression depth of lay rescuers in the intervention group was higher than in the original wording group (53.1 ± 11.4 mm vs. 46.7 ± 12.6 mm, *p* = 0.03) (Fig. 3). Mean complete chest release was 60.7% in the intervention group and 79.7% in the original wording group (*p* = 0.185). Correct hand position was performed by 87% of lay rescuers in the intervention group and 91% in the original wording group and did not differ (*p* = 0.946).

**Table 3:** CPR-Quality

Time			Check for breathing instruction					
			Original wording			Head tilt-chin lift instruction		
			MV ± SD		Percentage %, (n)	MV ± SD	Percentage %, (n)	*p*-value
Initial Original *n* = 14 Specified *n* = 27	Compression rate (bpm)	86.8 ± 15.8				102.4 ± 14.5		0.12
	Compression depth (mm)	46.8 ± 12.9				53.4 ± 11.6		0.019
	Correct chest release (n)			57.1% (8)			33.3% (9)	0.189
	Correct hand position (n)			92.9% (13)			88.9% (24)	1.00
0 – 1 Min Original *n* = 14 Specified *n* = 22	Compression rate (bpm)	87.4 ± 19.0				101.6 ± 13.6		0.089
	Compression depth (mm)	47.0 ± 13.0				54.1 ± 11.6		0.017
	Correct chest release (n)			71.4% (10)			45.5% (10)	0.176
	Correct hand position (n)			92.9% (13)			86.4% (19)	1.00
1 – 2 Min Original *n* = 12 Specified *n* = 13	Compression rate (bpm)	97.3 ± 19.6				107.2 ± 17.6		0.406
	Compression depth (mm)	45.9 ± 13.6				54.0 ± 11.6		0.046
	Correct chest release (n)			66.7% (8)			38.5% (5)	0.238
	Correct hand position (n)			91.7% (11)			84.6% (11)	1.00
2 – 3 Min Original *n* = 10 Specified *n* = 4	Compression rate (bpm)	95.8 ± 21.8				117.7 ± 19.4		0.142
	Compression depth (mm)	46.0 ± 9.6				55.7 ± 7.7		0.142
	Correct chest release (n)			60.0% (6)			50.0% (2)	1.00
	Correct hand position (n)			90.0% (9)			100.0% (4)	1.00
Total Original *n* = 14 Specified *n* = 27	Compression rate (bpm)	91.5 ± 17.2				103.1 ± 14.0		0.135
	Compression depth (mm)	46.7 ± 12.6				53.1 ± 11.4		0.030
	Correct chest release (n)			79.7%			60.7%	0.185
	Correct hand position (n)			91.0%			87.0%	0.946

## Discussion

### Quality of execution of T-CPR instructions

Significantly fewer lay rescuers (64.3%) performed a check for breathing in the original wording group in comparison with the specified instruction (92.6%) asking for head tilt-chin lift. In the intervention group the head tilt-chin lift manoeuvre was performed significantly more often and the time duration for check for breathing was appropriate. According to the ERC guidelines the check for breathing including looking, listening and feeling for breathing should take no more than 10 s [19]. The Bavarian T-CPR algorithm 2013 uses a similar wording as our modified check for breathing with the head tilt-chin lift instructions and showed a similar frequency of 91% [13]. The concretised check for breathing instruction used in simulations 21—48 fulfils the criteria of the ERC guidelines (lifting the chin to open the airway) significantly better. Based on the data, the primary study hypothesis of higher quality of check for breathing by the modified check for breathing instruction can be confirmed. Meanwhile the total time period of the emergency call is not significantly extended. To keep the instructions as simple as possible, the screening question "Is he/she breathing normally?" as recommended by the AHA seems useful [20]. To rule out communication shortfalls, specified instructions with description of execution should be included in the T-CPR protocol. For further improvement, regional differences in the description of gasping and agonal breathing should be considered, evaluated and implemented [21]. Bolle et al. and Yang et al. have shown that video-assisted telephone resuscitation can improve check for breathing and sufficient head tilting [22, 23]. New techniques such as video T-CPR can improve check for breathing. In a randomised controlled trial Ecker et al. showed a more guideline-compliant frequency and depth of chest compressions with the use of real-time video feedback systems compared to audio-only T-CPR instructions [24]. However, the immediate potential of optimising the wording of T-CPR protocols has not been fully realised yet.

The secondary study hypothesis of a prolonged time until start of chest compression with introduction of the modified wording could not be confirmed. The time interval from beginning of the emergency call to start of CPR was significantly longer with original wording (95 ± 33 s.) compared to the specified wording (80 ± 25 s.). Both groups started chest compression earlier than in a corresponding study of the Bavarian telephone resuscitation algorithm 2013 with 202 s [13]. Although the specified wording contained more words, it led to a faster start of chest compressions. One reason for this might be, that participants understood the instruction better and could therefore perform this task faster. In real emergency calls time intervals from the beginning of the emergency call to the start of chest compressions are significantly longer ranging from 167 to 229 s [20, 25, 26]. One reason for this may be that the simulated dispatcher knew what was going to happen and that no additional tasks such as alerting vehicles had to be performed. Kim and colleagues showed, that a higher number of incoming emergency calls to the emergency dispatch centre is associated with a delayed provision of T-CPR [27]. In this study the dispatcher answered only one emergency call per scenario, which further decreases transferability of the time intervals of this study. Additionally, in real-life incidents, factors such as the correct description of the patient's breathing, agonal breathing and the state of consciousness influence the time to first chest compression and the T-CPR instructions [28].

In comparison with the study by Klotz using the Bavarian telephone resuscitation protocol (46/46 = 100%) slightly fewer lay rescuers performed a consciousness check in our study (original wording group: 85.7% vs. intervention group 100%) [13]. The question “Is he/she awake?” may be the cause of the small discrepancy in the results. The question does not describe a specific action. The Bavarian telephone resuscitation algorithm 2013 contains the questions: “Does he/she react when you speak to him/her loudly? … if you touch him/her vigorously on the shoulder? “ [13]. The questions are much more detailed and include concrete actions [13]. There are no explicit recommendations on the wording neither from the ERC nor uniformly in German language. It appears that additional standardised phrases in case of misunderstanding of the initial question "Is he/she awake?" might be a useful addition to the telephone resuscitation protocol.

It can be noticed that a single lay rescuer who did not remove the clothing had already started CPR before the T-CPR instructions were given. There is no standardised wording for ongoing CPRs in the existing protocol. Hardeland and colleagues analysed data from the emergency dispatch centres of Copenhagen, Stockholm and Oslo and showed, that in cases, where CPR was already initiated, dispatchers were less likely to provide instructions [29]. However, as the skills and knowledge of bystanders are unknown, there remains potential to increase quality, if the dispatcher adds CPR instructions to the ongoing resuscitation. As the knowledge of the general public on providing CPR increases, it can be hoped that CPR attempts initiated prior to contacting the emergency dispatcher will rise as well. Alfsen et al. showed that cardiac arrest was sometimes not detected by the emergency dispatcher because of a deviation from the T-CPR protocol [30]. A strict adherence to protocol increases the detection rate of OHCA [30]. To keep the bystander motivated during the T-CPR and to reassess the quality of T-CPR execution, a separate protocol section focusing on feedback and encouragement should be developed and implemented [31]. Only three quarters of lay rescuers in both groups positioned themselves right-angled to the resuscitation simulator. This positioning is not specifically formulated in the instruction, which may be one of the reasons why fewer lay rescuers paid attention to this element. Positioning oneself orthogonal to the patient may be beneficial for correctly locating the pressure point in the centre of the chest. Adjustments of the position instruction may improve the orthogonality to the patient.

### Quality of CPR

The mean chest compression frequency did not differ significantly between both groups. Plata et al. also showed non-guideline compliant chest compression frequencies in a randomised controlled simulated T-CPR manikin trial with 78 bpm [31]. Even though the dispatcher counted the rhythm out loud at the beginning of chest compressions, lay rescuers did not reach the target frequency. In contrast, Klotz's study shows an average frequency of chest compressions of 107 bpm [13]. The reason for the more correct chest compression frequency may be the instruction to the lay rescuer to count out loud in the Bavarian T-CPR algorithm 2013 [13]. Kim et al. showed that the best CPR quality in terms of depth and frequency can be reached when lay rescuers count out loud and the dispatcher corrects this rate [27]. Including such an instruction in the T-CPR protocol might be a good alteration.

The average compression depth over the entire time was lower in the original wording group compared to the intervention group. The compression depth was not part of the study's hypothesis, so this significance may have been due to cumulative alpha error and missing Bonferroni correction.

Nearly all participants in both groups had a correct hand position. The wording of the T-CPR protocol "in the middle of the chest" is thus practicable and confirms the results of Lee et al. [32]. Hand position can be significantly improved with real-time video feedback during CPR [33]. Sufficient chest release did not differ significantly between original wording group and intervention group (79.7% vs. 60.7%). In contrast to compression frequency and depth, chest release can only be slightly improved using video feedback [24, 33]. In addition to these efforts, T-CPR protocol wording regarding chest release should be improved at the same time.

### Limitation

The foremost limitation results from the chosen study design, which is a non-randomized simulation trial. The present study was part of a large feasibility study. Due to this, randomisation was not possible. The study design as a non-randomised trial study is a limitation. We have to assume that mainly participants with primary interest in the topic came forward. Since participants were aware, that they would encounter a simulated cardiac arrest, an expectation bias with a potential positive effect on the execution quality must be considered. Participants were less surprised and most likely had lower stress levels. Due to study design the dispatcher knew, that the caller would describe a person in OHCA, which reduced the time until recognition of OHCA. Therefore, sensitivity and specificity of the algorithm regarding cardiac arrest recognition were not evaluated. Increasing the accuracy of OHCA recognition is a topic of ongoing research [34]. Another limitation is the small study population. Furthermore, the lay rescuers participating in this study were younger and proportionally more male than in cases of real OHCA [35]. It can be expected that younger rescuers are physically fitter and have a quicker response time. Another limitation is the simulated dispatcher, who was an emergency physician trained by a real dispatcher. However, the impact of this limitation is minimised by strictly following the T-CPR protocol. Furthermore, the CPR frequency was only counted at the beginning of the simulated emergency call and no continuous metronome was used. In some real dispatch centres the metronome is played to the caller constantly in most cases, which could have an impact on the frequency of chest compression. All communication between dispatcher and lay rescuer was in German. Translating instructions into English might have let to small semantic differences from the German version.

## Conclusions

Most of the instructions given in the T-CPR protocol were executed adequately. However, check of consciousness was a difficulty for the participants in the original wording group. With the specified wording including head tilt-chin lift instructions check for breathing was carried out significantly more adequately. It could be shown, that detailed instructions for check for breathing should be used to avoid difficulties in understanding. To improve chest compression frequency the dispatcher should continuously count out loud or transmit the clicking of a metronome. To reach the full potential of T-CPR wording of every T-CPR protocol should be evaluated regarding comprehension and practicability.

## Data Availability

The datasets used and analysed during the current study are available from the corresponding author on reasonable request.

## Abbreviations

T-CPR: Telephone cardiopulmonary resuscitation
CPR: Cardiopulmonary resuscitation
OHCA: Out of hospital cardiac arrest
ERC: European Resuscitation Council
AED: Automated external defibrillator
AHA: American Heart Association
